# Mitochondrial genome of *Aromia bungii* (Coleoptera: Chrysomeloidea: Cerambycidae) and phylogenetic analysis

**DOI:** 10.1080/23802359.2020.1846475

**Published:** 2021-01-13

**Authors:** Ruimin Li, Xiang Song, Yimin Du

**Affiliations:** a School of Life Sciences, Gannan Normal University, Ganzhou, China; b National Navel Orange Engineering and Technology Research Center, Ganzhou, China

**Keywords:** Cerambycidae, mitochondrial genome, *Aromia bungii*, phylogenetic analysis

## Abstract

The red-necked longhorn beetle *Aromia bungii* is a major pest of peach orchards. In this study, we sequenced and analyzed the complete mitochondrial genome (mitogenome) of *A. bungi,i*. This mitogenome was 15,760 bp long and encoded 13 protein-coding genes (PCGs), 22 transfer RNA genes (tRNAs) and two ribosomal RNA unit genes (rRNAs). Gene order was conserved and identical to most other previously sequenced Cerambycidae. Most PCGs of *A. bungii* have the conventional start codons ATN (six ATT, five ATG and one ATC), with the exception of *nad1* (TTG). Except for three genes (*cox1*, *cox2* and *nad5*) end with the incomplete stop codon T−, all other PCGs terminated with the stop codon TAA or TAG. The whole mitogenome exhibited heavy AT nucleotide bias (74.3%). Phylogenetic analysis positioned *A. bungii* in a well-supported clade within the subfamily Cerambycinae with *Xystrocera globosa* (tribe Xystrocerini). These results support the currently accepted taxonomy and provide a better understanding of the phylogenetic analysis of the Cerambycidae.

*Aromia bungii* (Faldermann) (Coleoptera: Cerambycidae) is a wood-boring pest of trees in the Rosaceae family, particularly peach, cherry, plum, apricot and pear (Men et al. 2019). This beetle is native to China, Mongolia, Korean Peninsula, Vietnam, Russia, and has been introduced into Germany, Italy, and Japan since 2011 (Fukaya et al. 2017). As *A. bungii* spends most of its life in a larval stage under the bark, chemical control with pesticides is labor-intensive, costly, and difficult. To get more information about this species, we sequenced and analyzed its mitogenome in this study.

Specimens of *A. bungii* were collected from Jingangshan City, Jiangxi Province, China (26°34′N, 114°05′E, July 2019) and were stored in Entomological Museum of Gannan Normal University (Accession number GNU-EAB09). Total genomic DNA was extracted from tissues using DNeasy DNA Extraction kit (Qiagen, Hilden, Germany). The mitogenome sequence of *A. bungii* was generated using Illumina HiSeq 2500 Sequencing System (Illumina, San Diego, CA). A total of 32.8 million reads were generated and had been deposited in the NCBI Sequence Read Archive (SRA) with accession number SRR12785341. Then, raw reads were assembled using MITObim v 1.7 (Hahn et al. 2013). By comparison with the homologous sequences of other Cerambycidae species from GenBank, the mitogenome of *A. bungii* was annotated using software GENEIOUS R11 (Biomatters Ltd., Auckland, New Zealand).

The complete mitogenome of *A. bungii* is 15,760 bp in length (GenBank accession no. MT371041), and containing the typical set of 13 protein-coding, two rRNA and 22 tRNA genes, and one non-coding AT-rich region. Gene order was conserved and identical to most other previously sequenced Cerambycidae (Li et al. 2016; Liu et al. 2018; Behere et al. 2019; Wang et al. 2019). The nucleotide composition of the mitogenome was biased toward A and T, with 74.3% of A + T content (A 39.8%, T 34.5%, C 14.8%, G 10.9%). Four PCGs (*nad4*, *nad4l*, *nad5* and *nad1*) were encoded by the minority strand (N-strand) while the other nine were located on the majority strand (J-strand). Most PCGs of *A. bungii* have the conventional start codons ATN (six ATT, five ATG and one ATC), with the exception of *nad1* (TTG). Except for three genes (*cox1*, *cox2* and *nad5*) end with the incomplete stop codon T−, all other PCGs terminated with the stop codon TAA or TAG. The 22 tRNA genes vary from 63 bp (*trnE*) to 71 bp (*trnK*). Two rRNA genes (*rrnL* and *rrnS*) locate at *trnL1*/*trnV* and *trnV*/control region, respectively. The lengths of *rrnL* and *rrnS* in *A. bungii* are 1282 and 781 bp, with the AT contents of 79.5% and 76.7%, respectively.

Phylogenetic analysis was performed based on the nucleotide sequences of 13 PCGs from 18 Coleoptera species. Phylogenetic tree was constructed through raxmlGUI 1.5 (Silvestro and Michalak 2012). Phylogenetic analysis positioned *A. bungii* in a well-supported clade within the subfamily Cerambycinae with *Xystrocera globosa* (tribe Xystrocerini) (Figure 1). The relationships ((Callichromatini + Xystrocerini) + ((Achrysonini + Clytini) + (Cerambycini + (Obriini + (Callipogonini + Prionini))))) were supported in Cerambycinae, and Cerambycinae was sister to Lamiinae within Cerambycidae.

**Figure 1.**
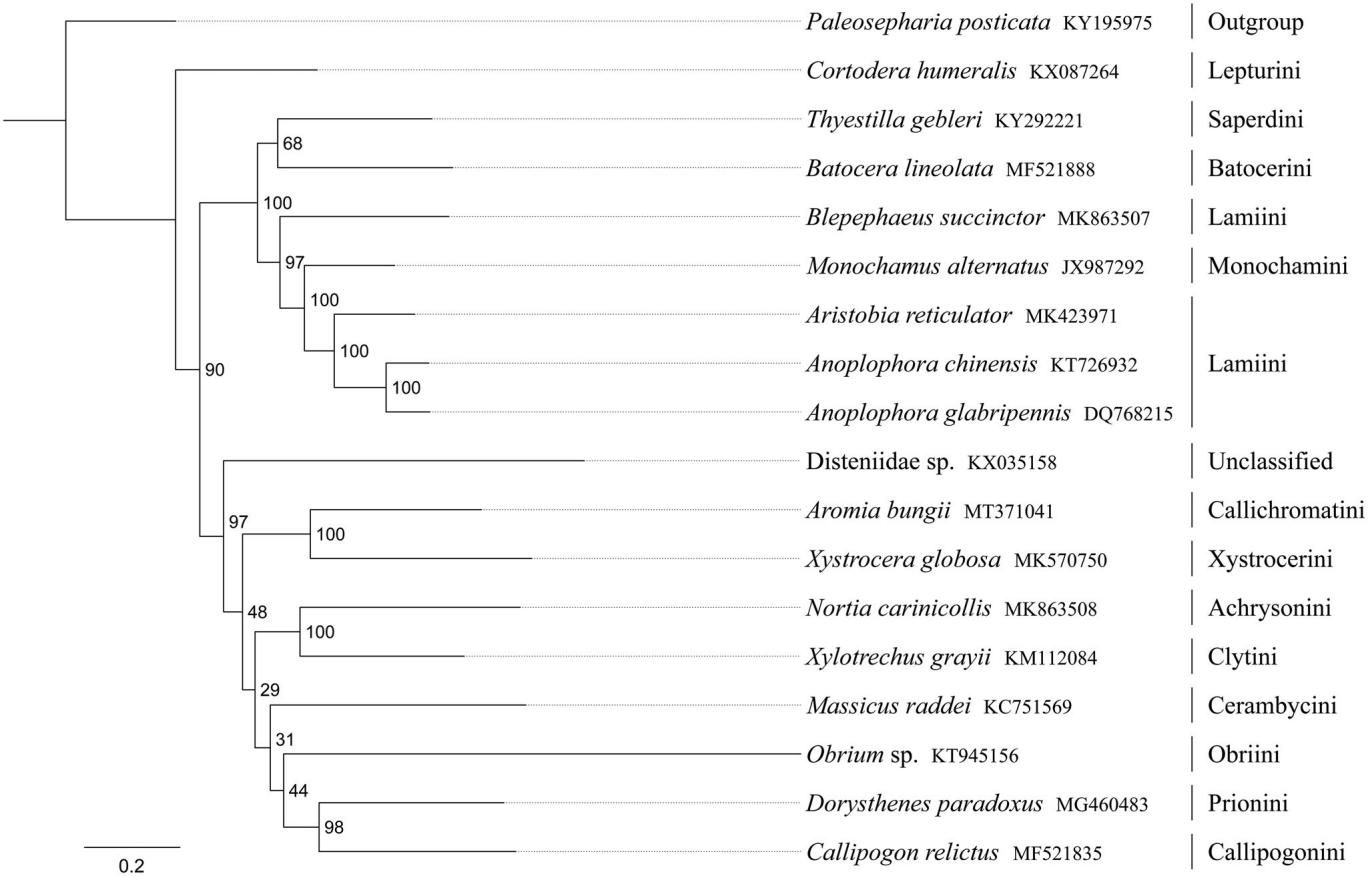
Phylogenetic relationships based on the 13 mitochondrial protein-coding genes sequences inferred from RaxML. Numbers on branches are bootstrap support values (BS).

## Data Availability

The data that support the findings of this study are openly available in NCBI (National Center for Biotechnology Information) at https://www.ncbi.nlm.nih.gov/, reference number MT371041, SRR12785341.

## References

[CIT0001] Behere GT, Tay WT, Firake DM, Kunz D, Burange PS, Ramamurthy VV. 2019. Characterization of draft mitochondrial genome of guava trunk borer, *Aristobia reticulator* (Fabricius, 1781) (Coleoptera: Cerambycidae: Lamiinae) from India. Mitochondrial DNA Part B. 4(1):1592–1593.

[CIT0002] Fukaya M, Kiriyama S, Yasui H. 2017. Mate-location flight of the red-necked longicorn beetle, *Aromia bungii* (Coleoptera: Cerambycidae): an invasive pest lethal to Rosaceae trees. Appl Entomol Zool. 52(4):559–565.

[CIT0003] Hahn C, Bachmann L, Chevreux B. 2013. Reconstructing mitochondrial genomes directly from genomic next-generation sequencing reads—a baiting and iterative mapping approach. Nucleic Acids Res. 41(13):e129–e129. 23661685 10.1093/nar/gkt371 PMC3711436

[CIT0004] Li W, Yang X, Qian L, An Y, Fang J. 2016. The complete mitochondrial genome of the citrus long-horned beetle, *Anoplophora chinensis* (Coleoptera: Cerambycidae). Mitochondrial DNA Part A. 27(6):4665–4667. 10.3109/19401736.2015.1106493 26643058

[CIT0005] Liu Y, Chen D, Liu H, Hu H, Bian H, Zhang R, Yang R, Jiang X, Shi S. 2018. The complete mitochondrial genome of the longhorn beetle *Dorysthenes paradoxus* (Coleoptera: Cerambycidae: Prionini) and the implication for the phylogenetic relationships of the Cerambycidae species. J Insect Sci. 18(2):21–29. 10.1093/jisesa/iey012 PMC5833319 29718483

[CIT0006] Men J, Zhao B, Cao D, Wang W, Wei J. 2019. Evaluating host location in three native *Sclerodermus* species and their ability to cause mortality in the wood borer *Aromia bungii* (Coleoptera: Cerambycidae) in laboratory. Biol Control. 134:95–102.

[CIT0007] Silvestro D, Michalak I. 2012. RaxmlGUI: a graphical front-end for RAxML. Org Divers E. 12(4):335–337.

[CIT0008] Wang J, Dai X, Xu X, Zhang Z, Yu D, Storey KB, Zhang J. 2019. The complete mitochondrial genomes of five longicorn beetles (Coleoptera: Cerambycidae) and phylogenetic relationships within Cerambycidae. PeerJ. 7(9):e7633. 31534857 10.7717/peerj.7633 PMC6732212

